# Supplementary figures and images for: Transcriptome and epigenome diversity and plasticity of muscle stem cells following transplantation
Source: PLoS Genet. 2020 Oct 30;16(10):e1009022. doi: 10.1371/journal.pgen.1009022 (PMC7657492; doi:10.1371/journal.pgen.1009022)

## Supplementary Figure 1

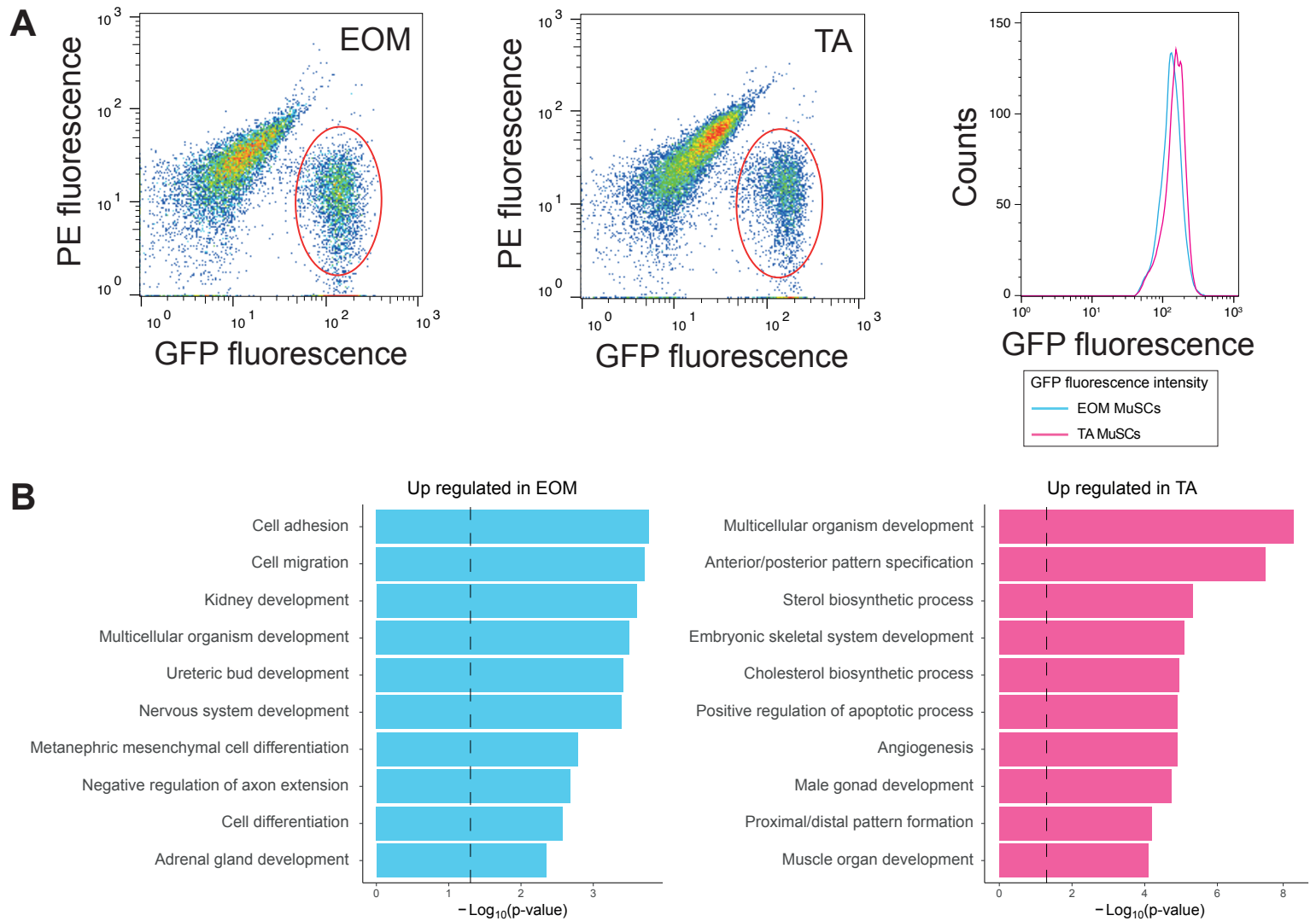

Supplement: S1 Fig — (A) FACS profiles of EOM (left) and TA (middle) dissociated muscles. GFP-positive MuSCs (regions outlined in red) were isolated. (right) Overlay of GFP fluorescence intensity of EOM and TA MuSCs. (B) Gene ontology analysis of the genes upregulated in EOM or TA MuSCs. The 10 most significant categories are shown. (PDF) [file pgen.1009022.s002.pdf]

Supplementary Figure 2

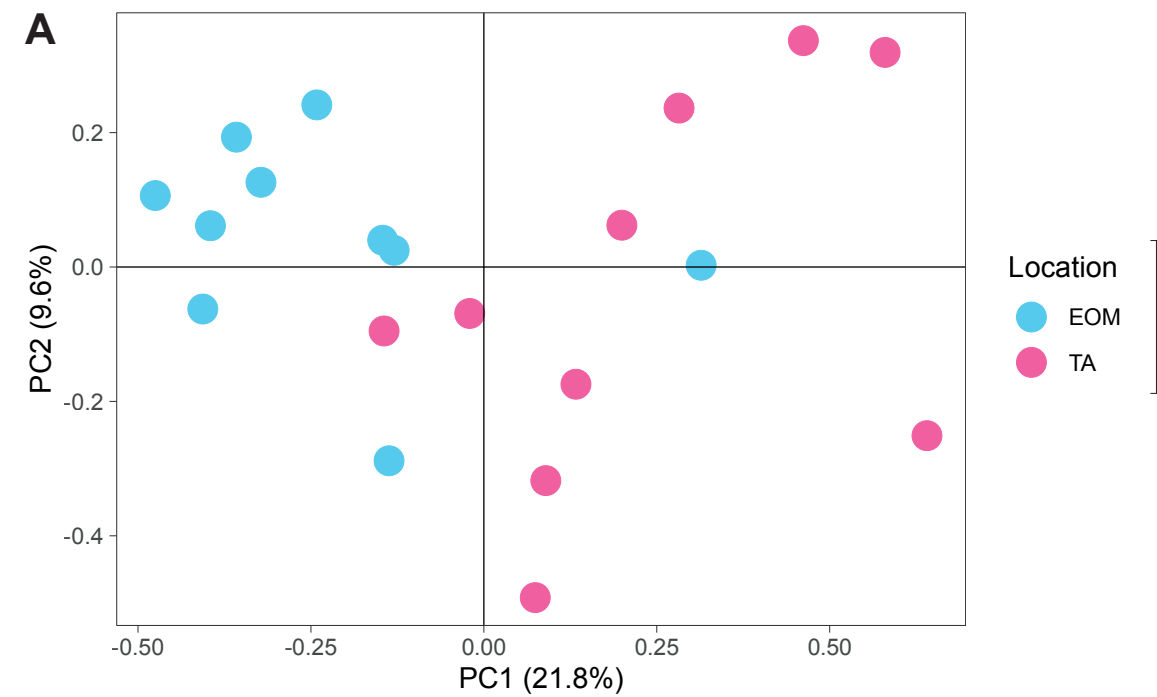

**B** Methylation TA vs EOM MuSCs (all enhancers)

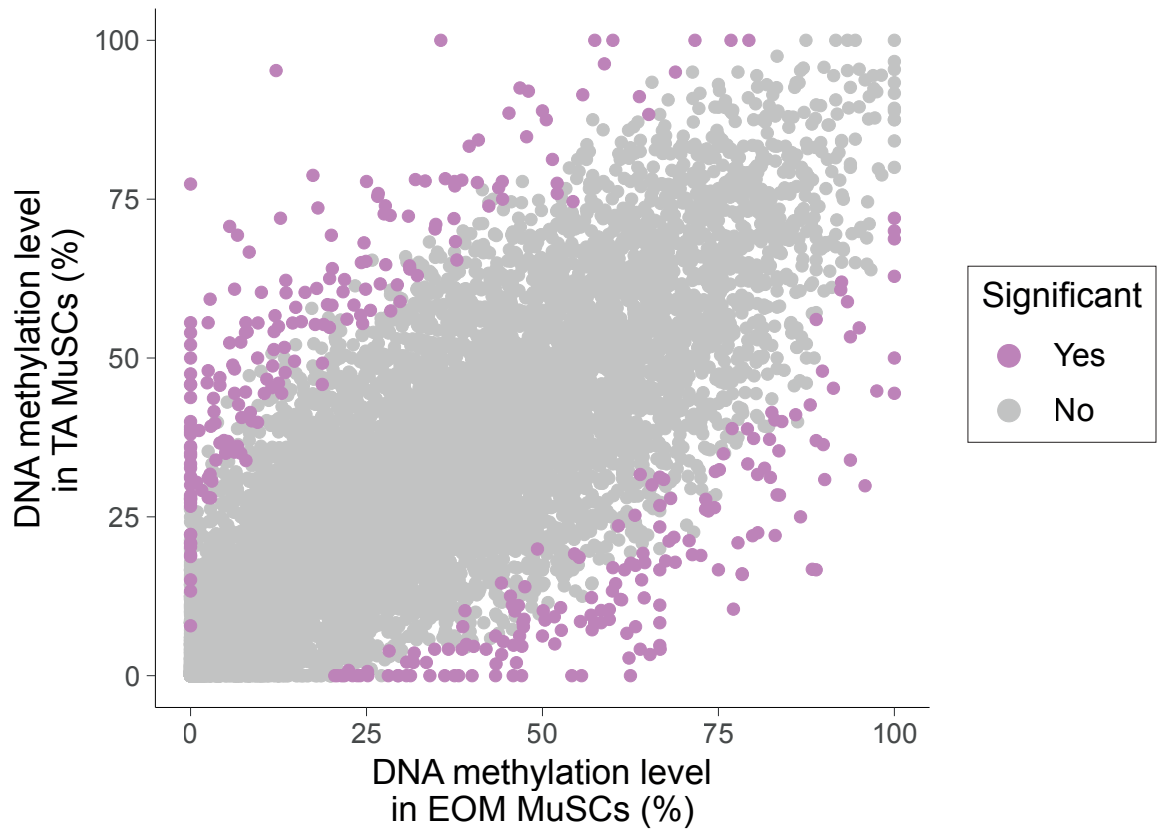

Supplement: S2 Fig — (A) PCA analysis of DNA methylation of 544 enhancers associated with genes specifically expressed in EOM or TA MuSCs. EOM and TA MuSCs separate according to their location. (B) Scatter plot comparing the mean DNA methylation levels of all enhancers in EOM and TA MuSCs. Enhancers were colour coded as significant if they were found to be differentially methylated by a rolling Z score approach (p < 0.05). See Fig 2E for genes with location-specific expression and enhancer methylation. (PDF) [file pgen.1009022.s003.pdf]

# Supplementary Figure 3

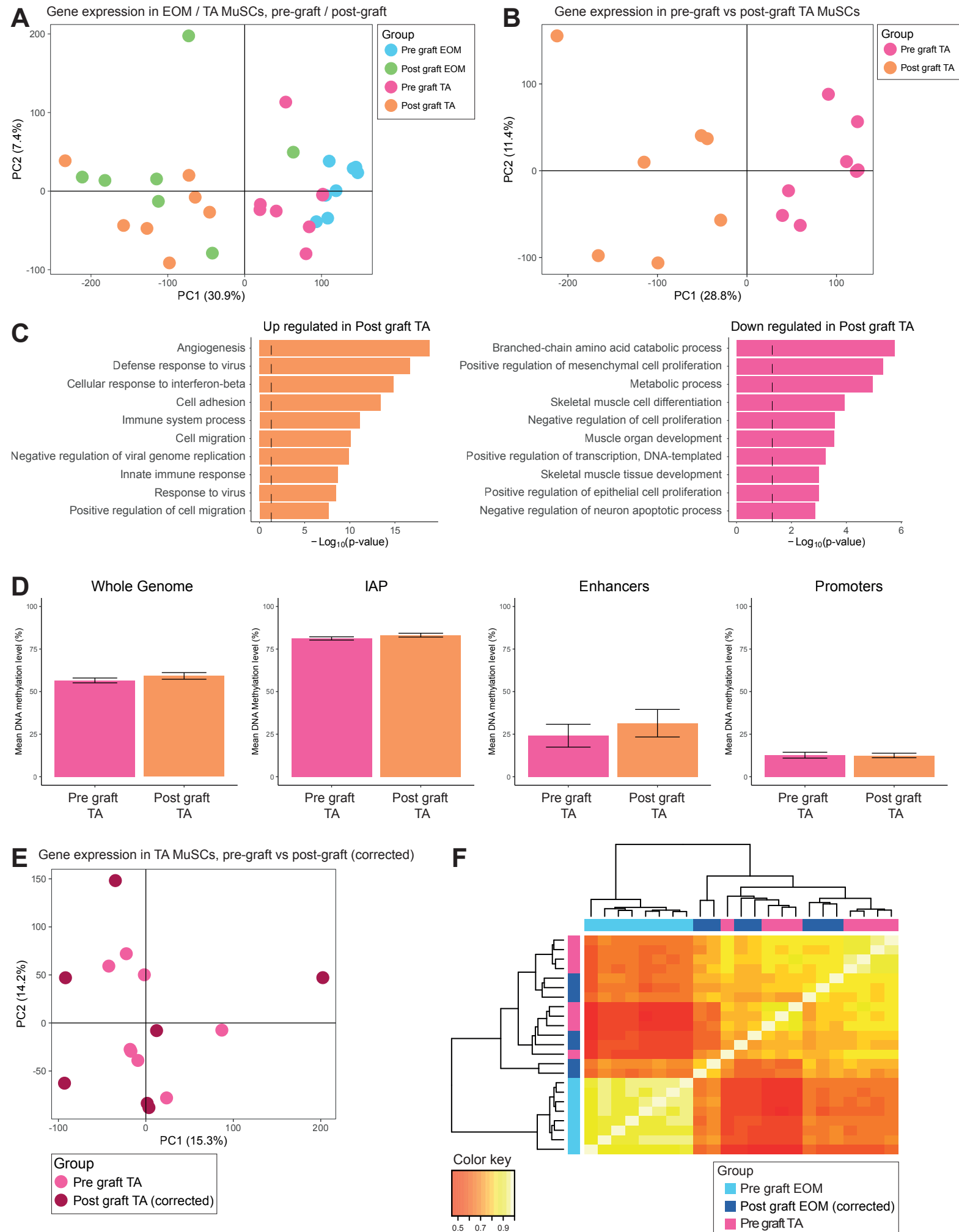

Supplement: S3 Fig — (A) Whole transcriptome PCA analysis of pre-graft and post-graft TA and EOM MuSCs. Samples separate into before and after grafting. (B) Whole transcriptome PCA analysis of pre-graft and post-graft TA MuSCs only. Pre and post-graft samples still cluster away from each other, indicating that the EOM samples were not solely responsible for the clustering in (A) and that there is a residual impact from the grafting procedure even after the recovery period. (C) GO categories of genes upregulated (left) and downregulated (right) in post-graft vs pre-graft TA MuSCs. Upregulated categories suggest there was a remaining inflammatory response. (D) Mean DNA methylation levels in pre-graft and post-graft TA MuSCs for whole genome, repeat elements, promoters and enhancers. Overall there was a global increase in DNA methylation after grafting. (E) Whole transcriptome PCA analysis of pre-graft TA MuSCs and post-graft MuSCs after applying a correction coefficient accounting for transcriptome modifications specifically induced by the transplantation procedure (see Methods). Pre and post-graft samples no longer cluster separately. (F) Hierarchical clustering analysis using Euclidean distance of Pearson correlation values between TA pre-graft, EOM pre-graft and EOM post-graft MuSCs. The samples cluster separately based on anatomical location. Notably, after engrafting EOM MuSCs into TA muscle they cluster with TA MuSCs rather than EOM MuSCs. (PDF) [file pgen.1009022.s004.pdf]

# Supplementary Figure 4

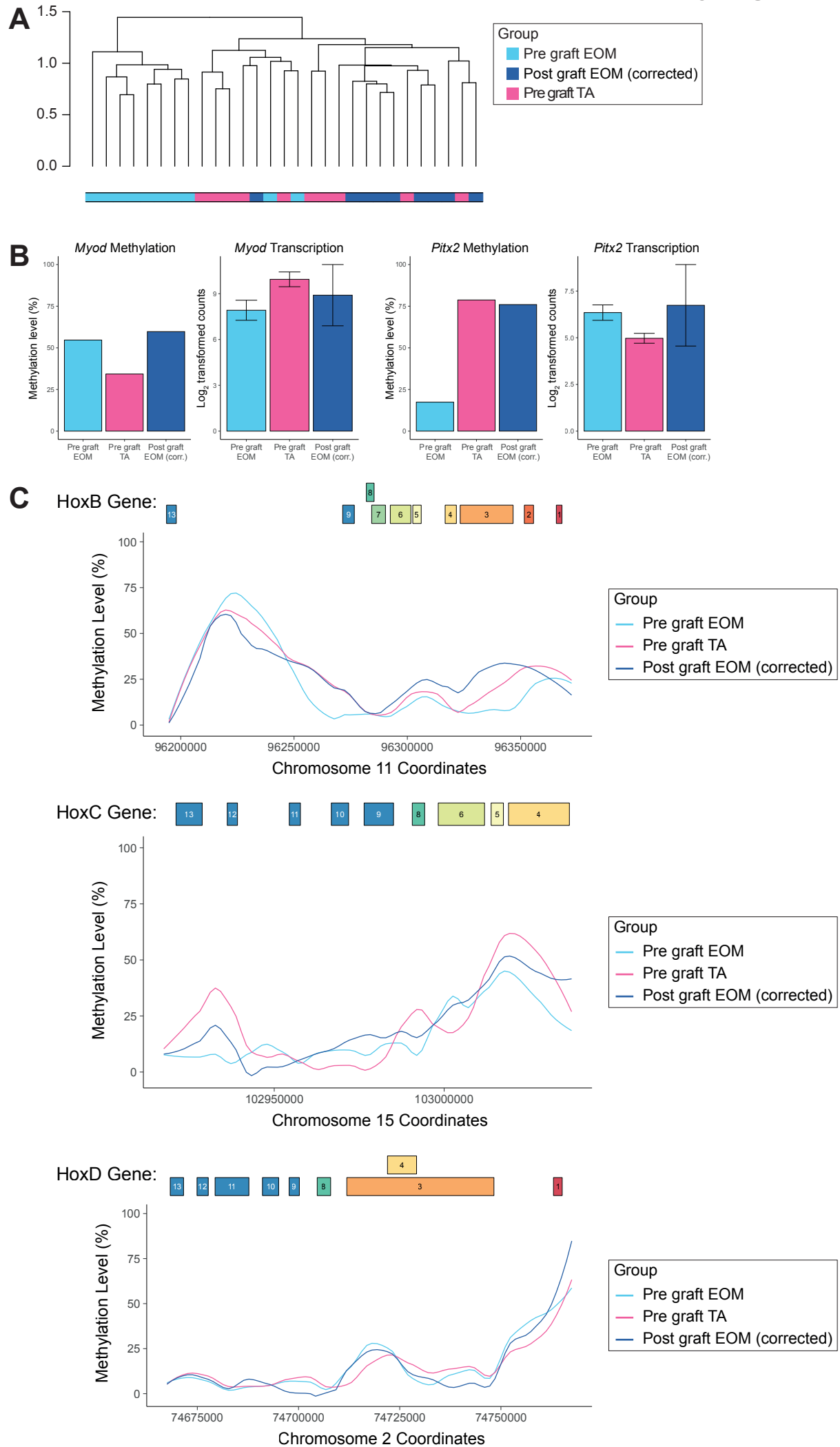

Supplement: S4 Fig — (A) Hierarchical clustering analysis using Euclidean distance of Pearson correlation values between TA pre-graft, EOM pre-graft and EOM post-graft MuSCs overall clusters samples based on location. Notably, post-graft EOM MuSCs cluster with TA MuSCs rather than with EOM MuSCs. (B) Enhancer methylation and gene expression levels of Myod and Pitx2 prior to and following grafting. (C) DNA methylation level across the HoxB (top), HoxC (middle) and HoxD (bottom) gene clusters in pre-graft EOM, pre-graft TA and post-graft EOM samples. DNA methylation levels were similar between EOM MuSCs and TA MuSCs. In addition, grafting EOM MuSCs into the TA muscle environment did not substantially affect the DNA methylation across these regions. (PDF) [file pgen.1009022.s005.pdf]
