# Supplementary material for: Transcriptome and epigenome diversity and plasticity of muscle stem cells following transplantation
Source: PLoS Genet. 2020 Oct 30;16(10):e1009022. doi: 10.1371/journal.pgen.1009022 (PMC7657492; doi:10.1371/journal.pgen.1009022)
Supplement: S1 Table — (PDF) [file pgen.1009022.s006.pdf]

			RNA seq											BS seq				
Sample	Cell number	Group	Passed QC	Reason for failing QC	Total Read Count	Forward Read Count	Reverse Read Count	Percent in Gene	Percent in exons	Percent in rRNA	Percent Genes Measured	Percent in MT	Percent on sense strand	Total Read Count	Forward Read Count	Reverse Read Count	Passed BSseq QC	Reason for failing BSseq QC
Pre_Graft_1_EOM_Exp1	500	EOM	No	Below total read count threshold	2526	1258	1268	90,18987744	75,38339131	0,755699498	2,919620808	8,110751254	50,10508028	3984478	2396772	1587706	Yes	
Pre_Graft_1_TA_Exp1	500	TA	No	Below percent in exons threshold	2694738	1348117	1346621	73,03559015	61,4123425	0,432921764	65,05616187	4,05988112	50,10284173	3124624	1837702	1286922	Yes	
Graft_1_EOM_Exp1	156	EOM to TA	No	Below percent in exons threshold	8118000	4056772	4061228	74,31045775	59,86262126	0,403978425	65,88066598	4,578246078	50,09315883	5240168	3173582	2066586	Yes	
Graft_1_TA_Exp1	43	TA to TA	No	Below percent in exons threshold	8118128	4064844	4053284	67,22523366	51,09128276	0,262776243	64,12809687	3,718075046	50,20200634	2618435	1546105	1072330	Yes	
Pre_Graft_3_EOM_Exp1	500	EOM	Yes		9714819	4855763	4859056	93,51584894	76,62899137	0,724236457	53,46929021	2,911528188	49,95224251	3262872	1959499	1303373	Yes	
Pre_Graft_3_TA_Exp1	500	TA	Yes		9308622	4649308	4659314	93,07168382	74,87022883	0,908354716	55,6639847	3,926426543	50,11751135	3693672	2010695	1682977	Yes	
Graft_3_EOM_Exp1	100	EOM to TA	No	Below percent in exons threshold	9836285	4921322	4914963	67,80642539	55,76630088	0,244547533	60,69863778	5,391259804	49,95964551	1916836	1121556	795280	Yes	
Graft_3_TA_Exp1	24	TA to TA	Yes		9865674	4931243	4934431	79,22706962	69,96656877	0,501624232	60,02549191	5,44851335	50,17973462	2944671	1766983	1177688	Yes	
Pre_Graft_4_EOM_Exp1	500	EOM	Yes		7838450	3916739	3921711	92,94604627	75,6263681	0,745339286	56,0742452	3,044337031	49,97531637	5724331	3399684	2324647	Yes	
Pre_Graft_4_TA_Exp1	500	TA	Yes		6582304	3290488	3291816	91,42184123	75,09392623	0,769314483	58,32072015	3,220825069	50,25973618	3471919	1924985	1546934	Yes	
Graft_4_EOM_Exp1	209	EOM to TA	Yes		10526422	5256309	5270113	88,28918632	71,36606746	0,227194044	51,41400462	3,848697414	49,95126555	895	504	391	No	Below total read count threshold
Graft_4_TA_Exp1	28	TA to TA	No	Below percent in exons threshold	6768072	3378817	3389255	70,67912877	56,06331452	0,294868973	58,31275392	4,104152234	50,08825938	1607720	962806	644914	Yes	
Pre_Graft_5_EOM_Exp1	500	EOM	Yes		9471491	4728932	4742559	93,58848502	75,55792704	0,994485877	54,45710189	3,643428772	50,15849241	3537638	2088006	1449632	Yes	
Pre_Graft_5_TA_Exp1	500	TA	No	Below total read count threshold	222822	111412	111410	91,19169941	78,77264236	0,526668098	36,25428184	2,53263049	49,88788362	12491074	7098514	5392560	Yes	
Graft_5_EOM_Exp1	105	EOM to TA	No	Below percent in exons threshold	9708205	4855043	4853162	76,02445475	62,1141577	0,286291	59,45192384	4,908080657	50,10830155	2329456	1369250	960206	Yes	
Graft_5_TA_Exp1	36	TA to TA	No	Below percent in exons threshold	1139937	569878	570059	70,97556352	61,13137404	0,278096141	48,43862025	4,859778773	49,70819874	2041521	1188335	853186	Yes	
Pre_Graft_1_EOM_Exp2	500	EOM	Yes		8580937	4288170	4292767	93,92541256	79,59085786	1,167448329	53,31793197	7,021739897	49,99451211	4299795	2619022	1680773	Yes	
Pre_Graft_1_TA_Exp2	500	TA	Yes		8888641	4462385	4426256	91,99653198	78,41363368	1,69199174	51,24273082	22,93827093	50,32893719	2429682	1368710	1060972	Yes	
Graft_1_EOM_Exp2	46	EOM to TA	Yes		5517157	2759657	2757500	84,72944087	74,93079003	0,56734565	56,27738389	15,89844554	50,1478624	3287164	1944096	1343068	Yes	
Graft_1_TA_Exp2	26	TA to TA	Yes		7373776	3706789	3666987	82,76502411	71,25249094	1,462189876	53,49318888	25,3478087	50,47282198	3744447	2230205	1514242	Yes	
Pre_Graft_2_EOM_Exp2	500	EOM	Yes		14402975	7204134	7198841	92,90538725	76,91790527	1,42060353	50,50585517	8,16326724	49,81367102	2971898	1774302	1197596	Yes	
Pre_Graft_2_TA_Exp2	500	TA	Yes		11372962	5683972	5688990	84,84251451	73,38443463	1,242364925	59,23683582	16,96431074	49,95241536	4383725	2485686	1898039	Yes	
Graft_2_EOM_Exp2	35	EOM to TA	Yes		9472455	4742871	4729584	89,4076115	80,9335573	1,242123349	42,61132797	33,03771331	50,06935284	2135945	1240777	895168	Yes	
Graft_2_TA_Exp2	67	TA to TA	Yes		9327101	4661691	4665410	89,88366732	73,80782874	0,658059026	48,48243448	16,42485708	50,08474135	2647527	1455104	1192423	Yes	
Pre_Graft_3_EOM_Exp2	500	EOM	No	Outlier in group	11093142	5539570	5553572	87,17112052	76,79700025	1,384625466	54,31370987	18,29608282	49,8062178	3118779	1872511	1246268	Yes	
Pre_Graft_3_TA_Exp2	500	TA	Yes		9855767	4915867	4939900	90,21110607	77,75402091	0,948175304	46,02485462	14,42974675	49,87659585	1454312	785157	669155	Yes	
Graft_3_EOM_Exp2	16	EOM to TA	Yes		6802637	3387031	3415606	91,70628905	80,12674953	9,438975386	47,16402454	59,62696	49,88199538	1492074	885602	606472	Yes	
Graft_3_TA_Exp2	16	TA to TA	Yes		5378534	2669422	2709112	75,52384841	73,83395154	1,453079311	61,79399347	46,83940504	49,70965776	2967698	1807125	1160573	Yes	
Pre_Graft_4_EOM_Exp2	500	EOM	Yes		13363659	6681346	6682313	93,2906331	74,95184777	0,872231419	54,8394806	4,331800788	50,27834165	9222445	5502069	3720376	Yes	
Pre_Graft_4_TA_Exp2	500	TA	Yes		3326270	1662095	1664175	93,02931719	78,78842211	0,464837902	51,820282	4,225485665	49,76042663	8557770	4775275	3782495	Yes	
Graft_4_EOM_Exp2	9	EOM to TA	Yes		7830185	3918610	3911575	80,02761509	72,92853341	1,302369123	55,03863618	37,06962635	50,1515974	2676672	1604720	1071952	Yes	
Graft_4_TA_Exp2	78	TA to TA	Yes		8258350	4112506	4145844	89,28080937	74,99759842	1,454942548	48,6975225	32,27851736	49,64247798	3425477	1920639	1504838	Yes	
Pre_Graft_5_EOM_Exp2	500	EOM	Yes		12301509	6143743	6157766	93,92563017	79,40682033	0,60683463	53,79988847	3,270604992	49,9812523	5723189	3372833	2350356	Yes	
Pre_Graft_5_TA_Exp2	500	TA	Yes		9026147	4528852	4497295	92,82411688	75,80510025	0,849375467	55,55245758	6,994724008	50,24110001	3781453	2112089	1669364	Yes	
Graft_5_EOM_Exp2	60	EOM to TA	Yes		5243925	2624030	2619895	83,32368228	77,60947927	2,348929483	58,04588545	45,33801121	49,98864409	4429479	2658831	1770648	Yes	
Graft_5_TA_Exp2	141	TA to TA	Yes		7345185	3688828	3656357	79,82365201	69,01545455	1,237329435	58,20919302	19,33845234	50,3848372	2705658	1559481	1146177	Yes	
Pre_Graft_6_EOM_Exp2	500	EOM	Yes		13514749	6760663	6754086	93,06693631	75,94415748	0,621321455	55,56839003	3,478315593	50,06965287	3875343	2322710	1552633	Yes	
Pre_Graft_6_TA_Exp2	500	TA	Yes		11938050	5967137	5970913	90,10236216	75,70691336	1,618095056	55,97466741	17,37997732	50,04611728	2646683	1481954	1164729	Yes	
Graft_6_EOM_Exp2	33	EOM to TA	Yes		8719716	4361861	4357855	77,47832187	68,66743098	1,2823181	60,9814387	24,83408691	50,08902471	4158705	2463848	1694857	Yes	
Graft_6_TA_Exp2	16	TA to TA	Yes		9360048	4677495	4682553	88,92941297	79,37538471	1,48382525	46,73783159	28,899288	50,00912706	2137529	1284880	852649	Yes	
