# Supplementary material for: Transcriptome and epigenome diversity and plasticity of muscle stem cells following transplantation
Source: PLoS Genet. 2020 Oct 30;16(10):e1009022. doi: 10.1371/journal.pgen.1009022 (PMC7657492; doi:10.1371/journal.pgen.1009022)
Supplement: Attachment — Submitted filename: Evano et al_PLOS Genetics_Response to Reviewers_24July2020.docx [file pgen.1009022.s007.docx]

We thank the Reviewers for their careful reading of our manuscript and for their many positive comments and detailed suggestions. We have now revised the manuscript according to the points raised by the Reviewers and our detailed point-by-point response is listed below.

**Summary of Revisions:**

**Text**: Revised text is highlighted in the marked-up version of the manuscript file, using the ‘Track Changes’ option in Microsoft Word.

**Fig 2A**: New representation of mean methylation levels distributions as violin plots.

**S1A Fig**: New FACS profiles of EOM and TA muscle stem cells showing consistency between sample types.

**Reviewer #1:** The manuscript by Evano et al. describes transcriptome and methylome differences between muscle stem cells from mouse hindlimb (TA) and extraocular muscles (EOMs) before and after transplantation into hindlimb muscles. Previous work from this group showed EOM muscle stem cells successfully engrafted into injured hindlimb muscle, but, based on expression of a small number of EOM-specific genes, failed to maintain the EOM muscle phenotype in their new location. They have followed this work up in the current paper by generating transcriptomes and DNA cytosine methylomes from TA and EOM muscle stem cells, pre- and post-engraftment into the TA. Consistent with their earlier work, EOM stem cells were reprogrammed after engraftment to a transcriptome resembling TA stem cells. A small percentage of EOM-specific genes were resistant to reprogramming, but the majority were highly responsive or of an intermediate character. The very interesting conclusion is that the local muscle microenvironment has a major role in dictating specific characteristics of stem cells associated with a given muscle, at a given body location.

We thank the Reviewer for thoroughly reading our manuscript and for pointing out the importance and novelty of our work.

The findings in this paper are straightforward and convincing. While it would be great if the reverse experiment could be performed (i.e., TA stem cells engrafted into the eye), this seems extremely difficult, if not impossible, with mice. My additional comments are minor.

1. It would be worth speculating on what potential differences between the TA and EOM environment could play a role in reprogramming during regeneration.

We thank the Reviewer for raising this point, which we have now addressed in the manuscript (Lines 304 to 308).

2. In Figure 3, two of the corrected post-graft EOM muscle samples are outliers, sitting on the 0 point for PC1. Can the authors comment on this?

We thank the Reviewer for raising this and have addressed this question in the manuscript (Lines 196 to 201).

3. On line 266, page 10, it is stated, “… indicating that stem cell-derived myogenic progenitors…”. It would avoid confusion to rephrase this as “… indicating that pluripotent stem cell-derived myogenic progenitors…”.

We thank the Reviewer for this comment, and we have now rephrased as suggested (Line 309).

**Reviewer #2:** In this manuscript, Evano et al. address the significant question of the differences between muscle satellite cells derived from distinct lineages (limb vs extraocular muscle) across multiple axes. In particular, the authors explore the molecular determinants of functional differences between limb and EOM satellite cells by identifying transcriptional and epigenetic signatures that differ between them and determining the extent to which these signatures are cell-intrinsic or determined by their surrounding niche.

Data presented here are novel additions to knowledge in the field, particularly the whole-genome epigenetic profiling of quiescent satellite cells. In addition, the discovery that gene expression and methylation patterns can be largely redetermined by niche factors in cross-transplanted cells suggests an intriguing degree of plasticity.

We appreciate the Reviewers' careful reading of the manuscript and for highlighting the interest of our work.

The figures are nicely clear and convey the experimental results logically. Some comments:

Figure 2A - As presented, the differential methylation data are somewhat underwhelming.

We appreciate this point raised by the Reviewer. We have now provided mean methylation levels across promoters, enhancers, CpG islands and IAP repeat elements as violin plots for each sample. While the conclusions appear similar, these plots are a higher resolution presentation of methylation level distributions and homogeneity across samples.

Figure 2E - While it is not unexpected in experiments of this sort, it would be nice if the authors would address genes (such as Adamtsl1) characterized by both significant over- and under-methylation when comparing limb and EOM-derived cells.

We thank the Reviewer for this comment; we have discussed this observation further in the manuscript (Lines 144 to 149).

Figure 3F - It would assist in interpretation if this panel also included data from pre-grafted TA and EOM satellite cells. If the data are already in-hand, inclusion of results from homologously grafted cells (TA at least) would also improve the impact of the figure/interpretation.

We thank the Reviewer for this comment. The data from pre-grafted TA and EOM MuSCs is already present in Fig 1E. We agree that it would facilitate interpretation of Fig 3F to have it side-by-side with Fig 1E, but we decided not to overload Fig 3. We have now linked Fig 3F to Fig 1E in the figure legend (Lines 452 to 453). In addition, the data corresponding to homologously grafted cells is presented in Fig 3F, right. We have modified the Figure legend accordingly to avoid confusion (Line 450).

The main concern I have with the writing is the perhaps excessive speculation/overinterpretation of some aspects of the results in the Discussion section, although this may be a matter of personal taste. However, in the context of this interesting if speculative discussion the lack of follow up for any of the genes identified and discussed as potential key determinants of the phenotype is somewhat of a missed opportunity.

We thank the Reviewer for raising this point. We agree that it would have been interesting to follow up on the genes we identified and assess their role in the regenerative capacity of EOM and TA MuSCs. However, this coincided with the time of lockdown due to COVID-19 and such experiments could not be initiated.
